# Upadacitinib in patients from China, Brazil, and South Korea with rheumatoid arthritis and an inadequate response to conventional therapy

**DOI:** 10.1111/1756-185X.14235

**Published:** 2021-11-15

**Authors:** Xiaofeng Zeng, Dongbao Zhao, Sebastiao C. Radominski, Mauro Keiserman, Chang K. Lee, Sebastian Meerwein, Jeffrey Enejosa, Yunxia Sui, Mohamed‐Eslam F. Mohamed, Won Park

**Affiliations:** ^1^ Department of Rheumatology Peking Union Medical College Hospital (PUMCH) Chinese Academy of Medical Sciences Beijing China; ^2^ Key Laboratory of Rheumatology & Clinical Immunology National Clinical Research Center for Dermatologic and Immunologic Diseases (NCRC‐DID) Beijing China; ^3^ Department of Rheumatology and Immunology Shanghai Changhai Hospital Shanghai China; ^4^ Department of Rheumatology Universidade Federal do Paraná Curitiba Brazil; ^5^ Department of Rheumatology Pontifical Catholic University Porto Alegre Brazil; ^6^ Rheumatology Asan Medical Center Seoul South Korea; ^7^ Immunology AbbVie Deutschland GmbH & Co. KG Ludwigshafen Germany; ^8^ Immunology AbbVie Inc. Chicago Illinois USA; ^9^ Rheumatology School of Medicine Inha University Incheon South Korea

**Keywords:** autoinflammatory conditions, biologic therapies, immunosuppressants, inflammation, rheumatoid arthritis

## Abstract

**Aim:**

This study assessed the efficacy and safety of upadacitinib (UPA), in combination with conventional synthetic disease‐modifying antirheumatic drugs (csDMARDs), in Chinese, Brazilian, and South Korean patients with active rheumatoid arthritis (RA) and an inadequate response (IR) to csDMARDs.

**Methods:**

Patients on stable csDMARDs were randomized (1:1) to once‐daily UPA 15 mg or matching placebo (PBO) for a 12‐week, double‐blind period. The primary endpoint was the proportion of patients achieving ≥20% improvement in American College of Rheumatology criteria (ACR20) at week 12.

**Results:**

In total, 338 patients were randomized and treated, of whom 310 (91.7%) completed the double‐blind phase. The study met the primary endpoint of ACR20 at week 12 for UPA 15 mg vs PBO (71.6% vs 31.4%, *P* < .001), with a treatment difference observed as early as week 1. All ranked and other key secondary endpoints, including more stringent responses such as ACR50, ACR70 (≥50%/70% improvement in ACR criteria), and Disease Activity Score in 28 joints using C‐reactive protein <2.6, were met for UPA 15 mg vs PBO. The incidence of serious infections (2.4% vs 0.6%) and herpes zoster (HZ: 1.8% vs 0.6%) was higher with UPA 15 mg vs PBO. There was one case of venous thromboembolism reported in the UPA group.

**Conclusion:**

UPA 15 mg in combination with csDMARDs demonstrated clinical and functional improvement and an acceptable safety profile over 12 weeks among patients from China, Brazil, and South Korea who had moderately to severely active RA and an IR to csDMARDs.

## 1 INTRODUCTION

Rheumatoid arthritis (RA) is a chronic systemic autoimmune disease that, if left untreated or inadequately treated, could lead to progressive functional impairment, significant disability, reduced quality of life, and increased mortality.^1^, ^2^ Methotrexate (MTX; a conventional synthetic disease‐modifying antirheumatic drug [csDMARD]) is generally the recommended first‐line therapy in the treatment of RA, with addition of other csDMARDs, biologic DMARDs (bDMARDs), or targeted synthetic DMARDs (tsDMARDs) in patients with an inadequate response (IR) after 3 months.^3^, ^4^, ^5^

There remains a large unmet medical need in the treatment of RA despite major progress over the last 30 years and development of therapies such as anti‐tumor necrosis factor, anti‐interleukin‐6, CTLA4‐Ig, and anti‐CD20 agents, among others.^6^, ^7^, ^8^, ^9^ The percentage of patients with RA who reach and maintain a status of low disease activity (LDA) or clinical remission (CR) remains unsatisfactory and, over time, many patients discontinue treatment due to adverse events (AEs) or loss of efficacy.^10^, ^11^ To address this, novel therapies are required to complement the available RA armamentarium.^10^, ^11^, ^12^

The Janus kinase (JAK) family of signaling molecules (JAK1, JAK2, JAK3, and tyrosine kinase 2 [TYK2]) mediates intracellular signaling downstream of multiple cytokines and growth factors.^13^ JAK pathway activation initiates the expression of survival factors and other molecules that facilitate leukocyte cell trafficking and proliferation, and thereby contributes to the pathogenesis of inflammatory and autoimmune disorders including RA.^13^, ^14^ Inhibition of JAK signaling is an established approach for the treatment of RA,^14^, ^15^, ^16^, ^17^ and JAK inhibitors form the tsDMARD class of treatments.^5^ Upadacitinib (UPA) is a JAK inhibitor engineered to have greater selectivity for JAK1 over JAK2, JAK3, and TYK2, and is approved by the United States Food and Drug Administration, the European Medicines Agency, the Pharmaceuticals and Medical Devices Agency, and several other regulatory agencies (including in South Korea and Brazil) for the treatment of patients with moderately to severely active RA and an IR to MTX.^18^, ^19^, ^20^, ^21^ UPA has a favorable benefit–risk profile based on several global phase III trials in a variety of patient populations.^22^, ^23^, ^24^, ^25^, ^26^

The objective of this study was to assess the efficacy and safety of UPA 15 mg in combination with csDMARDs over 12 weeks in patients from China, Brazil, and South Korea who had moderately to severely active RA and an IR to csDMARDs.

## 2 PATIENTS AND METHODS

### 2.1 Study design and participants

This is a phase III, multicenter study that includes 2 periods. This report describes the results from period 1, which was the 12‐week, randomized, double‐blind, placebo (PBO)‐controlled period of the study conducted at 37 sites in China, Brazil, and South Korea. Period 2 is the open‐label, 52‐week extension in patients who completed period 1 and which is ongoing and therefore not discussed in this report (Figure S1).

Eligible patients were adults (≥18 years) who had moderately to severely active RA and an RA diagnosis of ≥3 months duration, and who fulfilled the 2010 American College of Rheumatology/European League Against Rheumatism (ACR/EULAR) classification criteria for RA.^27^ Active disease was defined as ≥6 swollen joints (based on a swollen joint count of 66 joints [SJC66]) and ≥6 tender joints (based on a tender joint count of 68 joints [TJC68]) at screening and baseline visits, and a high‐sensitivity C‐reactive protein (CRP) concentration of ≥3 mg/L at screening. Patients had been receiving csDMARD therapy for ≥3 months and had been on a stable dose for ≥4 weeks prior to the first dose of study drug. Patients had a prior IR to ≥1 csDMARD (MTX, sulfasalazine, or leflunomide). Patients who had an IR to hydroxychloroquine and/or chloroquine were only included if they had also failed MTX, sulfasalazine, or leflunomide treatment. Prior exposure to ≤1 bDMARD for RA was allowed in up to 20% of patients if they had limited exposure (<3 months) or did not tolerate the bDMARD, and patients had to have discontinued bDMARD therapy prior to the first dose of study drug and gone through an appropriate washout period. Exclusion criteria included prior exposure to any JAK inhibitor, IR to bDMARD therapy, history of any arthritis with onset prior to age 17, or current diagnosis of inflammatory joint disease other than RA. Clinical tests at screening included chest X‐ray, electrocardiogram, tuberculin purified protein derivative skin test, hepatitis testing, and a serum pregnancy test. Patients with a history of any malignancy except for successfully treated non‐melanoma skin cancer (NMSC) or localized carcinoma in situ of the cervix were excluded from participating in the trial. All possible malignancies were identified using the search criteria “malignancies” Standard MedDRA Queries (SMQ) (narrow). Preferred terms which represent confirmed malignancies were subsequently identified based on a narrower “malignant tumors” SMQ.

The study was conducted according to the International Conference on Harmonization of Technical Regulations for Pharmaceuticals for Human Use guidelines, applicable regulations, and the Declaration of Helsinki. All study‐related documents were approved by independent ethics committees and institutional review boards. All patients provided written informed consent.

### 2.2 Randomization and masking

Patients who met the eligibility criteria were randomized 1:1 to receive either a once‐daily extended‐release formulation of UPA 15 mg or matching PBO, administered orally for 12 weeks, along with background csDMARD treatment. Randomization was stratified by country; patients from China were expected to comprise up to 80% of the total study population. Patients were randomized using an interactive response technology with a randomization schedule generated by the Data and Statistical Sciences Department of the study sponsor. Patients, investigators, and the sponsor were masked to this allocation. UPA 15 mg extended‐release tablets and PBO tablets were identical in appearance in order to maintain blinding.

### 2.3 Outcomes

The primary endpoint was the proportion of patients achieving ≥20% improvement in ACR criteria (ACR20 response) at week 12. Ranked key secondary endpoints at week 12 were change from baseline in Disease Activity Score in 28 joints using CRP (DAS28‐CRP), Health Assessment Questionnaire‐Disability Index (HAQ‐DI), and Short‐Form 36‐item Health Survey (SF‐36), and the proportion of patients achieving LDA based on DAS28‐CR) ≤3.2, CR based on DAS28‐CRP <2.6, and LDA based on Clinical Disease Activity Index (CDAI) ≤10 (see Table S1). Other key secondary endpoints were the proportion of patients achieving an ACR50/70 response (≥50%/70% improvement in ACR criteria) at week 12 and an ACR20 response at week 1 (see Table S1). Additional endpoints included change from baseline in pain using a visual analog scale, remission based on CDAI ≤2.8, and Boolean remission (defined as SJC [based on 28 joints] ≤1, TJC [based on 28 joints] ≤1, CRP ≤1 mg/dL, and patient's global assessment of disease activity ≤10 mm [range: 0‐100 mm]). Blood samples for pharmacokinetic analysis were obtained throughout the study. AEs, physical examinations, laboratory assessments, electrocardiograms, and vital signs data were assessed throughout the study.

### 2.4 Statistical analysis

A sample size of 322 was planned to provide ≥90% power for a 21.7% difference in ACR20 response rate at week 12 (assuming a PBO ACR20 response rate of 36.7%), at a 2‐sided significance level of 0.05 and accounting for a 10% dropout rate. This sample size was also planned to provide ≥90% power for most of the key secondary endpoints, including change from baseline in DAS28‐CRP, ACR50 response rate, LDA and CR based on DAS28‐CRP, and SF‐36 Physical Component Summary (PCS), at a 2‐sided significance level of 0.05 and accounting for a 10% dropout rate.

All efficacy analyses were carried out using the Full Analysis Set (FAS), which included all randomized patients who received ≥1 dose of study drug. For binary endpoints, frequencies and percentages were reported for each treatment group and comparison between UPA 15 mg and PBO was conducted using the Cochran–Mantel–Haenszel test adjusting for the stratification factor (country). Non‐responder imputation was used to handle missing data for binary endpoints. Patients who discontinued the study drug prematurely were considered as non‐responders for all subsequent visits after discontinuation, and patients with missing values at a specific visit were considered as non‐responders for that visit. For the continuous endpoints of change from baseline in DAS28‐CRP and HAQ‐DI, missing data were handled by multiple imputation (MI) and statistical inference was conducted using analysis of covariance (ANCOVA), with treatment group as the fixed factor and the corresponding baseline value and country as covariates. For other continuous endpoints, statistical inference was conducted using the mixed‐model repeated measures (MMRM) method, which included the fixed effects of treatment, visit, treatment by visit interaction, and country, and the fixed covariate of baseline value in the model, using an unstructured variance–covariance matrix. From both the ANCOVA (coupled with MI) and MMRM analyses, the least squares (LS) mean and 95% confidence interval (CI) were reported for each treatment group, and LS mean treatment differences and associated 95% CI and *P* values were reported comparing UPA 15 mg with PBO. A sequential testing method was used to control the overall type I error rate of primary and ranked key secondary endpoints.

Safety analyses were carried out using the Safety Analysis Set, which included all patients who received ≥1 dose of study drug. Patients with treatment‐emergent AEs (TEAEs) were tabulated by preferred term as in the Medical Dictionary for Regulatory Activities, system organ class, severity, and relationship to study drug as assessed by the investigator.

This trial was registered with ClinicalTrials.gov, identifier: NCT02955212.

## 3 RESULTS

### 3.1 Patients

**FIGURE 1:**
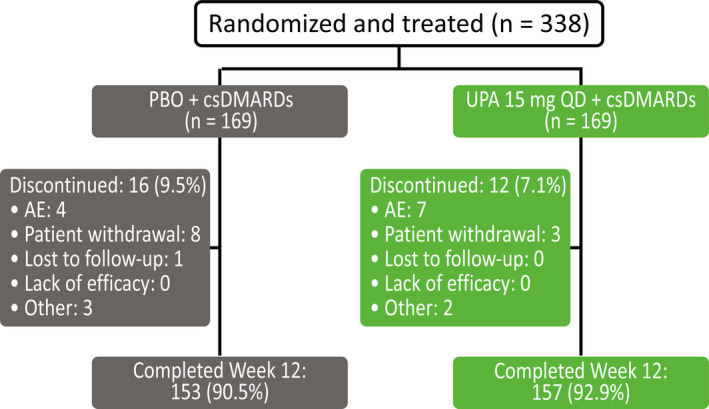
Patient disposition. AE, adverse event; csDMARD, conventional synthetic disease‐modifying antirheumatic drug; PBO, placebo; QD, once daily; UPA, upadacitinib

Between January 3, 2018 and August 14, 2019, 338 patients from 37 sites in China, Brazil, and South Korea were randomized to UPA 15 mg (n = 169) or PBO (n = 169). Discontinuation rates through week 12 were similar in the UPA 15 mg and PBO groups, although discontinuation due to AEs was more common in the UPA 15 mg group and patient withdrawal of consent was more common in the PBO group (Figure 1). All 338 patients were included in the FAS.

At baseline, demographics and disease characteristics were generally well balanced across the UPA and PBO groups; 228 (67.5%), 58 (17.2%), and 52 (15.4%) patients were enrolled in China, South Korea, and Brazil, respectively. Patients from each country were split evenly between UPA 15 mg and PBO. Most patients were female (81.1%) with a mean age of 51.7 years in both the UPA 15 mg and PBO groups. Patients had a mean (SD) disease duration of 7.2 (7.2) and 7.5 (7.6) years in the UPA 15 mg and PBO groups, respectively. All patients were on background csDMARDs for at least 3 months, and at least 4 weeks on stable doses prior to the first dose of study drug. Subjects had to have failed (lack of efficacy) at least 1 of the following: MTX, sulfasalazine, or leflunomide. Subjects with IR to hydroxychloroquine and/or chloroquine could only be included if they had also failed (lack of efficacy or intolerability) MTX, sulfasalazine, or leflunomide. Up to week 24, the background csDMARD dose had to be kept stable and could be decreased only for safety reasons. The majority of patients received either MTX alone or MTX in combination with another csDMARD, and most patients also received stable treatment with low‐dose oral glucocorticoids (Table 1). The concomitant csDMARDs the patients were receiving are detailed in Table S2.

**TABLE 1:** Baseline demographics and disease characteristics

Characteristic, mean (SD)^a^	PBO +csDMARDs (n = 169)	UPA 15 mg QD +csDMARDs (n = 169)
Female, n (%)	139 (82.2)	135 (79.9)
Age, y	51.7 (11.4)	51.7 (10.6)
RA duration since diagnosis, y	7.5 (7.6)	7.2 (7.2)
Country, n (%)		
China	114 (67.5)	114 (67.5)
Brazil	26 (15.4)	26 (15.4)
South Korea	29 (17.2)	29 (17.2)
RF+ and/or anti‐CCP+, n (%)	152 (89.9)	159 (94.1)
TJC68	23.0 (14.5)	21.5 (14.8)
SJC66	11.9 (6.0)	11.9 (6.9)
DAS28‐CRP^b^	5.6 (0.9)	5.6 (1.0)
CDAI^c^	35.9 (11.2)	35.2 (12.4)
HAQ‐DI^b^	1.4 (0.7)	1.3 (0.7)
SF‐36 PCS^b^	34.2 (7.6)	34.5 (7.7)
Pain VAS^d^	63.8 (20.6)	66.8 (20.6)
CRP, mg/L	20.2 (25.2)	20.0 (21.5)
Prior bDMARD exposure, n (%)	3 (1.8)	5 (3.0)
csDMARD use at baseline		
MTX alone, n (%)	71 (42.0)	79 (47.0)
MTX and other csDMARD, n (%)	41 (24.3)	32 (19.0)
csDMARD other than MTX, n (%)	57 (33.7)	57 (33.9)
MTX dose^e^, mg/wk	13.0 (3.5)	12.9 (4.3)
Oral glucocorticoid use, n (%)	112 (66.3)	108 (63.9)
Mean glucocorticoid dose, mg/d^f^	6.1 (2.6)	5.5 (2.3)

Abbreviations: Anti‐CCP+, anti‐cyclic citrullinated peptide positive; bDMARD, biologic disease‐modifying antirheumatic drug; CDAI, Clinical Disease Activity Index; CRP, C‐reactive protein; csDMARD, conventional synthetic disease‐modifying antirheumatic drug; DAS28‐CRP, Disease Activity Score in 28 joints using CRP; HAQ‐DI, Health Assessment Questionnaire‐Disability Index; MTX, methotrexate; PBO, placebo; PCS, Physical Component Summary; QD, once daily; RA, rheumatoid arthritis; RF+, rheumatoid factor positive; SF‐36, Short‐Form 36‐item Health Survey; SJC66, swollen joint count of 66 joints; TJC68, tender joint count of 68 joints; UPA, upadacitinib; VAS, visual analog scale.

^a^ Unless otherwise stated.

^b^ PBO: n = 166, UPA: n = 166.

^c^ PBO: n = 166, UPA: n = 163.

^d^ PBO: n = 166, UPA: n = 165.

^e^ PBO: n = 112, UPA: n = 111.

^f^ PBO: n = 112, UPA: n = 108.

### 3.2 Efficacy

**FIGURE 2:**
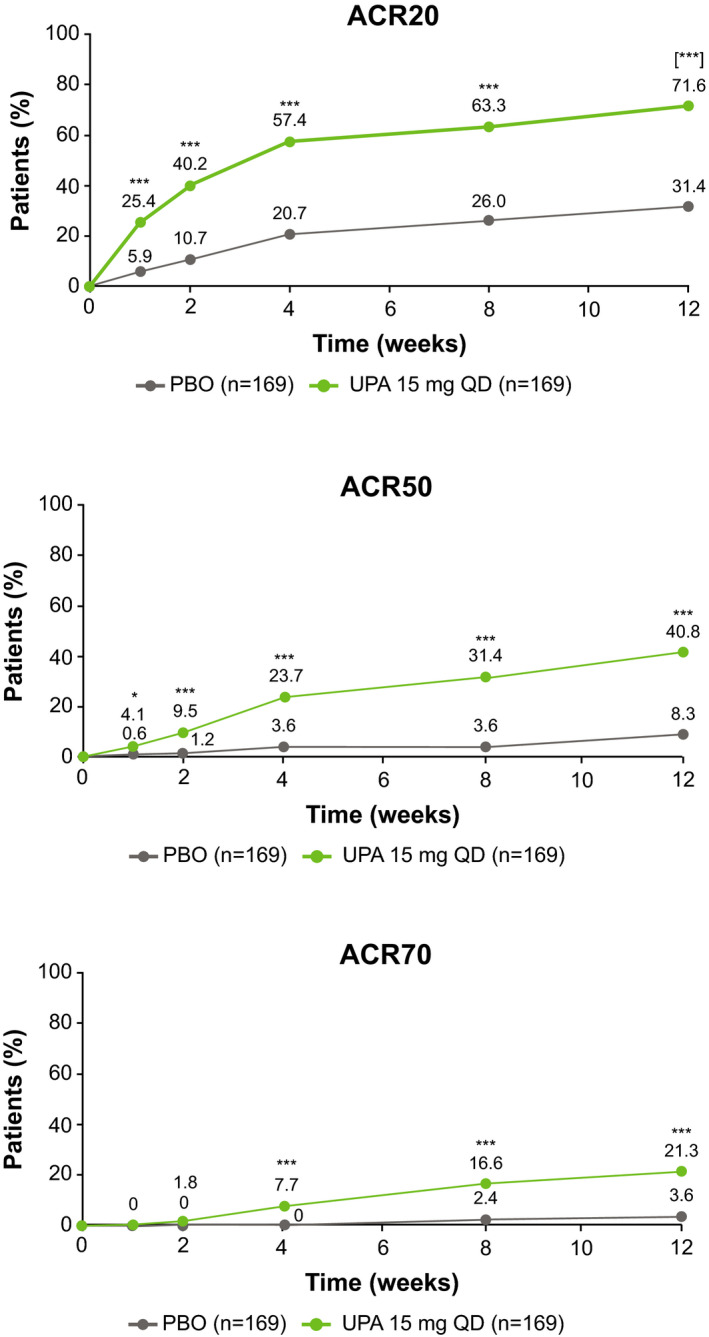
Proportion of patients achieving ACR responses over time (NRI). *Nominal *P* < .05 vs PBO; ***nominal *P* <.001 vs PBO (not adjusted for multiplicity); [***]*P* < .001 vs PBO (adjusted for multiplicity). ACR, American College of Rheumatology; ACR20/50/70, ≥20%/50%/70% improvement in ACR criteria; NRI, non‐responder imputation; PBO, placebo; QD, once daily; UPA, upadacitinib

At week 12, ACR20 (primary endpoint) was achieved by a significantly greater proportion of patients receiving UPA 15 mg vs PBO (71.6% [95% CI 64.8‐78.4] vs 31.4% [95% CI 24.4‐38.4], *P* < .001) (Figure 2). ACR50 and ACR70 responses were also achieved by greater proportions of patients receiving UPA 15 mg (40.8% and 21.3%) vs PBO (8.3% and 3.6%) at week 12 (nominal *P* < .001 for both comparisons) (Figure 2). Onset of action with UPA 15 mg was rapid, with 25.4% vs 5.9% of patients achieving ACR20 at week 1 with UPA 15 mg vs PBO, respectively (nominal *P* < .001) (Figure 2). A breakdown of the ACR20 placebo response and the ACR20 UPA 15 mg response by country is shown in Table 2, where South Korea had lower responses compared with China and Brazil. A breakdown of the ACR components is shown in Table S3, and demonstrates that greater improvements are seen with UPA 15 mg vs PBO in all components of the ACR response. In the UPA 15 mg group, ACR20 at week 12 was achieved by numerically greater proportions of patients receiving concomitant MTX and another csDMARD (78.1%) compared with MTX alone (72.2%) or a csDMARD other than MTX (68.4%). ACR20 response rates at week 12 by concomitant csDMARD at baseline are shown in Table S4. The differences in ACR20 response rates at week 12 between the treatment groups are as follows: for those on MTX only, 37%; for those on MTX and another csDMARD, 46.4%; and for those on a csDMARD other than MTX, 42.1%.

**TABLE 2:** Breakdown in ACR20 PBO response and ACR20 UPA 15 mg QD response for the 3 countries at week 12

	Responder, n (%)	
	PBO	UPA 15 mg QD
China, n = 114	36 (31.6)	82 (71.9)
South Korea, n = 29	7 (24.1)	19 (65.6)
Brazil, n = 26	10 (38.5)	20 (76.9)

Abbreviations: ACR20, ≥20% improvement in American College of Rheumatology criteria; PBO, placebo; QD, once daily; UPA, upadacitinib.

**FIGURE 3:**
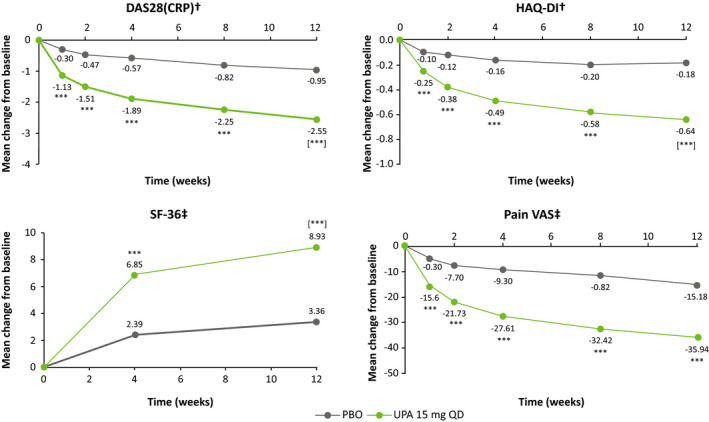
Mean change from baseline in DAS28‐CRP, HAQ‐DI, SF‐36 PCS, and pain VAS over time. ***Nominal *P* < .001 vs PBO (not adjusted for multiplicity); [***]*P* < .001 vs PBO (adjusted for multiplicity). ^†^Analysis of covariance coupled with multiple imputation. ^‡^Mixed‐model repeated measures. DAS28‐CRP, Disease Activity Score in 28 joints using C‐reactive protein; HAQ‐DI, Health Assessment Questionnaire‐Disability Index; PBO, placebo; PCS, Physical Component Summary; QD, once daily; SF‐36, Short‐Form 36‐item Health Survey; UPA, upadacitinib; VAS, visual analog scale

**FIGURE 4:**
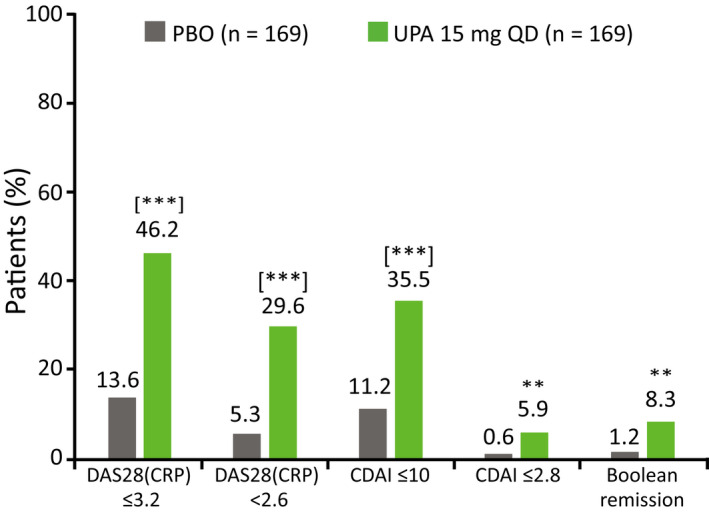
Proportion of patients achieving DAS28‐CRP ≤3.2/<2.6, CDAI ≤10/≤2.8, and Boolean remission at week 12 (NRI). **Nominal *P* < .01 vs PBO (not adjusted for multiplicity); [***]*P* < .001 vs PBO (adjusted for multiplicity). Boolean remission was defined as SJC (based on 28 joints) ≤1, TJC (based on 28 joints) ≤1, CRP ≤1 mg/dL, and patient's global assessment of disease activity ≤10 mm (range: 0‐100 mm). CDAI, Clinical Disease Activity Index; CRP, C‐reactive protein; DAS28‐CRP, Disease Activity Score in 28 joints using CRP; NRI, non‐responder imputation; PBO, placebo; QD, once daily; SJC, swollen joint count; TJC, tender joint count; UPA, upadacitinib

At week 12, all ranked secondary endpoints were met. Patients receiving UPA 15 mg showed significantly greater improvements from baseline vs PBO in DAS28‐CRP, HAQ‐DI, and SF‐36 PCS (Figure 3). LDA, as defined by DAS28‐CRP ≤3.2 and CDAI ≤10, was achieved by a significantly greater proportion of patients receiving UPA 15 mg compared with PBO at week 12 (Figure 4). CR, as defined by DAS28‐CRP <2.6, was also achieved by a significantly greater proportion of patients receiving UPA 15 mg compared with PBO at week 12 (Figure 4). Patients receiving UPA 15 mg also achieved greater response rates for more stringent measures of efficacy compared with PBO at week 12, including CDAI ≤2.8 and Boolean remission (Figure 4). In addition, at all visits from week 1 onward, improvements from baseline in all ACR components were greater (nominal *P* < .01) in the UPA 15 mg group compared with the PBO group, including improvements in patients’ assessments of pain (Figure 3).

### 3.3 Safety

Through week 12, rates of TEAEs were numerically higher in the UPA 15 mg group compared with the PBO group (Table 3). The most frequently reported TEAEs (≥2% of patients in any treatment group) through week 12 were upper respiratory tract infection (UPA 15 mg: 9.5%, PBO: 6.5%) and increased alanine aminotransferase (UPA 15 mg: 5.3%, PBO: 1.2%). The percentage of TEAEs leading to discontinuation of study drug through week 12 was numerically higher in the UPA 15 mg group compared with the PBO group (Table 3).

**TABLE 3:** Treatment‐emergent AEs through week 12

Event, n (%)	PBO+ csDMARDs (n = 169)	UPA 15 mg QD +csDMARDs (n = 169)
Any AE	83 (49.1)	104 (61.5)
Any SAE^b^	5 (3.0)	12 (7.1)
Any AE leading to discontinuation of study drug	5 (3.0)	8 (4.7)
Deaths^c^	0	0
AESI		
Serious infection^d^	1 (0.6)	4 (2.4)
Opportunistic infection	0	2 (1.2)
Latent/active tuberculosis	0	0
HZ	1 (0.6)	3 (1.8)
Hepatic disorder	12 (7.1)^a^	16 (9.5)
Gastrointestinal perforation	0	0
Renal dysfunction	0	0
Any malignancy, excluding NMSC	0	1 (0.6)
NMSC	0	0
MACE, adjudicated^e^	0	0
VTE, adjudicated^f^	0	1 (0.6)
Anemia	4 (2.4)	5 (3.0)
Neutropenia	0	5 (3.0)^b^
Lymphopenia	2 (1.2)	1 (0.6)
CPK elevation	1 (0.6)	3 (1.8)^c^

Abbreviations: AE, adverse event; AESI, AE of special interest; CPK, creatine phosphokinase; csDMARD, conventional synthetic disease‐modifying antirheumatic drug; DVT, deep vein thrombosis; HZ, herpes zoster; MACE, major adverse cardiovascular event; NMSC, non‐melanoma skin cancer; PBO, placebo; QD, once daily; SAE, serious AE; UPA, upadacitinib; VTE, venous thromboembolism.

^a^ SAEs were reported in no more than 1 patient in any treatment group, with the exception of pneumonia and tendon rupture.

^b^ Including non‐treatment‐emergent deaths.

^c^ Includes 4 cases of pneumonia (UPA: 3, PBO: 1). Also includes 1 case of HZ infection in the UPA group (also counted under HZ); the patient subsequently discontinued treatment.

^d^ Defined as cardiovascular death, non‐fatal myocardial infarction, and non‐fatal stroke.

^e^ Including DVT and pulmonary embolism; VTE observed in a patient with a history of DVT.

^f^ One patient on PBO experienced an event of drug‐induced liver injury and met Hy's law criteria.

^g^ Only Grade 1 or 2 decreases in neutrophil levels.

^h^ Grade 3 increases in CPK observed in 2 patients; neither had rhabdomyolysis.

The percentage of patients with serious AEs (SAEs) through week 12 was also numerically higher in the UPA 15 mg group compared with the PBO group (Table 3). Individual SAEs were reported in no more than 1 patient in either treatment group, except for pneumonia (3 cases in the UPA 15 mg group) and tendon rupture (2 cases in the UPA 15 mg group). SAEs leading to discontinuation of study drug were reported in 5 patients in the UPA 15 mg group (HZ, pneumonia, tendon rupture, worsening RA, and angioedema) and 2 patients in the PBO group (drug‐induced liver injury and pneumonia). For all but 1 patient (tendon rupture), the event resolved following discontinuation of the study drug and appropriate treatment. There were no patient deaths during the study.

Through week 12, the frequency of AEs of special interest (AESIs) in the UPA 15 mg group was generally similar compared with the PBO group, with the exception of serious infection, HZ, hepatic disorder, neutropenia, and creatine phosphokinase (CPK) elevation, which were reported in a higher percentage of patients in the UPA 15 mg group (Table 3). There were 5 serious infections (4 in the UPA 15 mg group and 1 in the PBO group) and HZ was reported in 4 patients (3 in the UPA 15 mg group and 1 in the PBO group); 1 patient in the UPA 15 mg group had a serious HZ event that led to discontinuation of study drug. Treatment‐emergent opportunistic infections were reported in 2 patients in the UPA 15 mg group (non‐serious cytomegalovirus infection and a non‐serious oral candidiasis). Treatment‐emergent malignancy was reported in 1 Korean patient in the UPA 15 mg group who was diagnosed with breast cancer on the day of randomization. This event led to discontinuation but was not considered by the investigator to have a reasonable possibility of being related to the study drug, but rather related to age (46 years) and/or environmental factors (former smoker and drinker). Pulmonary embolism and deep vein thrombosis (DVT) (both adjudicated by an external adjudication committee to be a venous thromboembolism [VTE]) were both reported in 1 patient in the UPA 15 mg group who had several risk factors for VTE, including obesity, a history of DVT, and use of estrogen preparations. There were no cases of active/latent tuberculosis, gastrointestinal perforation, renal dysfunction, treatment‐emergent NMSC, or adjudicated major adverse cardiovascular event in either treatment group through week 12.

Laboratory measures were assessed at baseline and at each study visit. The frequency of laboratory abnormalities was generally similar in both treatment groups, except for neutropenia and CPK elevation, which were reported in a higher percentage of patients in the UPA 15 mg group. No patients with CPK elevation had symptoms of muscle pain or rhabdomyolysis, or discontinued the study drug due to the elevation of CPK. Through week 12, hematology variables (hemoglobin, hematocrit, lymphocytes, neutrophils, and platelets) were generally within the normal range at baseline and at all visits for both treatment groups. Grade 3 and Grade 4 decreases in hematology values were generally infrequent and similar in both treatment groups. Grade 3 decreases in lymphocytes, from baseline through week 12, occurred frequently during the study, but were comparable between UPA 15 mg (16 patients [9.6%]) and PBO (17 patients [10.2%]) groups (Table S5). One patient in the PBO group experienced an event of drug‐induced liver injury and met Hy's law criteria. The patient was taking isoniazid as a prophylactic treatment for latent tuberculosis identified at screening, and the event resolved on day 45 after both isoniazid and study drug (PBO) were permanently discontinued.

### 3.4 Pharmacokinetics

Within 24 hours of dosing, UPA mean plasma concentrations ranged from 58.7 ng/mL (around the peak time) to 6.1 ng/mL (close to the trough time) in patients in the UPA 15 mg group. These concentrations were consistent with the predicted concentrations based on prior pharmacokinetic evaluations of UPA.^28^, ^29^

## 4 DISCUSSION

This study was the first to assess the efficacy and safety of UPA 15 mg in combination with csDMARDs in patients from China, Brazil, and South Korea with moderately to severely active RA and an IR to csDMARDs. The primary endpoint was met in this study, with a significantly greater proportion of patients who received UPA 15 mg achieving an ACR20 response at week 12 compared with those receiving PBO. All ranked key secondary endpoints (change from baseline in DAS28‐CRP, HAQ‐DI, SF‐36 PCS, LDA based on DAS28‐CRP, CR based on DAS28‐CRP, and LDA based on CDAI) showed a similar, clinically meaningful, and statistically significant improvement in patients receiving UPA 15 mg compared with patients receiving PBO. The efficacy results of this study were comparable with those of the SELECT‐NEXT study, which assessed the efficacy and safety of UPA in a global population of patients with RA and an IR to csDMARDs.^22^ In this study, similar to SELECT‐NEXT and SELECT‐COMPARE, all patients were on background csDMARDs for at least 3 months, and at least 4 weeks on stable doses prior to the first dose of study drug. In line with SELECT‐NEXT and SELECT‐COMPARE no changes in background treatment of concomitant csDMARDs were allowed during the double‐blind period of the study.

Treatment targets of LDA and CR are recommended by treat‐to‐target guidelines^4^, ^5^ and, in this study, more than one‐third of patients who received UPA 15 mg met LDA criteria as defined by DAS28‐CRP ≤3.2 and CDAI ≤10 after 12 weeks. Treatment with UPA 15 mg also improved the proportion of patients achieving stringent response criteria such as DAS28‐CRP <2.6, CDAI ≤2.8, and Boolean remission. RA has diverse detrimental effects on patients’ physical and mental well‐being,^30^, ^31^ and LDA and remission are associated with improvements in health‐related quality of life and physical function.^32^ In this study, improvements in physical function, as shown by patient‐reported outcomes including HAQ‐DI and SF‐36 PCS, were significantly greater in patients receiving UPA 15 mg compared with patients receiving PBO. The safety profile of this study is generally comparable with that of global studies, with no new safety signals observed in the Chinese, Brazilian, and South Korean population.^22^, ^23^

UPA 15 mg was generally well tolerated, although the proportion of patients with TEAEs, SAEs, and TEAEs leading to discontinuation of study drug was numerically higher in patients receiving UPA 15 mg compared with patients receiving PBO. The frequency of AESIs experienced by patients receiving UPA 15 mg through week 12 was generally similar compared with patients receiving PBO, except for serious infection, HZ, hepatic disorder, neutropenia, and CPK elevation, which were reported in a higher proportion of patients receiving UPA 15 mg. These events have also been reported in global phase III trials of UPA.^22^, ^24^, ^26^

This study has some limitations. The majority of patients were Chinese, so conclusions for patients from Brazil and South Korea may be limited. Structural damage was not assessed, therefore it was not possible to evaluate radiographic inhibition in response to UPA, as has been demonstrated in global studies.^23^, ^26^ Also, 12 weeks is a short period of time for safety analysis; the ongoing 52‐week open‐label extension will provide a more comprehensive report on safety.

In summary, UPA 15 mg in combination with csDMARDs demonstrated clinical and functional improvement and an acceptable safety profile among patients from China, Brazil, and South Korea who had moderately to severely active RA and an IR to csDMARDs. The benefit–risk profile of UPA 15 mg, based on efficacy and safety, was favorable in this patient population and was comparable with other studies in the UPA global phase III program.^22^, ^23^, ^24^, ^25^, ^26^

## CONFLICT OF INTEREST

SM is an employee of AbbVie Deutschland GmbH & Co. KG, and JE, M‐EM, and YS are employees of AbbVie Inc and may own stock or stock options. The other authors have declared no conflicts of interest.

## AUTHOR CONTRIBUTIONS

All authors were involved in drafting the article or revising it critically for important intellectual content, and all authors approved the final version to be submitted for publication. XZ had full access to all of the data in the study and takes responsibility for the integrity of the data and the accuracy of the data analysis. Study conception and design: CKL, SM, JE, YS, M‐EM; acquisition of the data: XZ, DZ, SR, MK, CKL, WP; analysis and interpretation of the data: XZ, DZ, SR, MK, CKL, SM, JE, YS, M‐EM, WP.

## Supporting information

Supplementary Material

## Data Availability

AbbVie is committed to responsible data sharing regarding the clinical trials we sponsor. This includes access to anonymized, individual, and triallevel data (analysis datasets), as well as other information (eg protocols and Clinical Study Reports), provided the trials are not part of an ongoing or planned regulatory submission. This includes requests for clinical trial data for unlicensed products and indications. These clinical trial data can be requested by any qualified researchers who engage in rigorous, independent scientific research, and will be provided following review and approval of a research proposal and statistical analysis plan, and execution of a Data Sharing Agreement. Data requests can be submitted at any time and the data will be accessible for 12 months, with possible extensions considered. For more information on the process, or to submit a request, visit https://www.abbvie.com/our‐science/clinical‐trials/clinical‐trials‐data‐and‐information‐sharing/data‐and‐information‐sharing‐with‐qualified‐researchers.html.
