# Supplementary material for: Upadacitinib in patients from China, Brazil, and South Korea with rheumatoid arthritis and an inadequate response to conventional therapy
Source: Int J Rheum Dis. 2021 Nov 15;24(12):1530–9. doi: 10.1111/1756-185X.14235 (PMC9299142; doi:10.1111/1756-185X.14235)
Supplement: Supplementary file 1 — Supplementary Material [file APL-24-1530-s001.docx]

# SUPPLEMENTARY MATERIAL

**
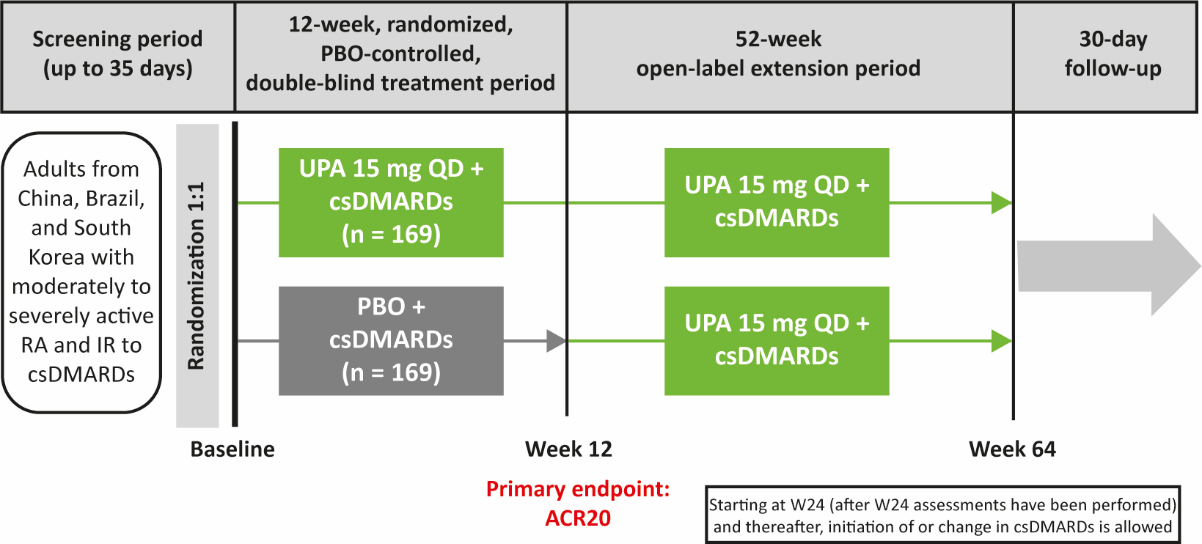
**

**Supplementary Figure 1** Study design. Abbreviations: ACR20, ≥20% improvement in American College of Rheumatology criteria; csDMARD, conventional synthetic disease-modifying antirheumatic drug; IR, inadequate response; PBO, placebo; QD, once daily; RA, rheumatoid arthritis; UPA, upadacitinib; W, week

**supplementary table 1** Statistical hierarchy

| Primary endpoint (at Week 12) | ACR20 |
| --- | --- |
| Ranked key secondary efficacy analyses (at Week 12) | 1. Change from baseline in DAS28(CRP)  2. Change from baseline in HAQ-DI  3. Change from baseline in SF-36 PCS  4. Proportion of subjects achieving LDA based on DAS28(CRP) ≤3.2  5. Proportion of subjects achieving CR based on DAS28(CRP)  6. Proportion of subjects achieving LDA based on CDAI ≤10 |

Abbreviations: ACR20/50/70, ≥20%/50%/70% improvement in American College of Rheumatology criteria; CDAI, Clinical Disease Activity Index; CR, clinical remission; DAS28(CRP), Disease Activity Score in 28 joints using C-reactive protein; HAQ-DI, Health Assessment Questionnaire-Disability Index; LDA, low disease activity; PCS, Physical Component Summary; SF-36, Short-Form 36-item Health Survey

**Supplementary Table 2** Concomitant csDMARDs and oral corticosteroids by generic name

|  | PBO (n = 169) | UPA 15 mg QD (n = 169) | Total (n = 338) |
| --- | --- | --- | --- |
| Any csDMARD | 169 (100) | 168 (99.4) | 337 (99.7) |
| Chloroquine | 0 | 1 (0.6) | 1 (0.3) |
| Hydroxychloroquine | 51 (30.2) | 34 (20.1) | 85 (25.1) |
| Leflunomide | 52 (30.8) | 51 (30.2) | 103 (30.5) |
| Meropenem | 1 (0.6) | 0 | 1 (0.3) |
| Methotrexate | 112 (66.3) | 111 (65.7) | 223 (66.0) |
| Sulfasalazine | 20 (11.8) | 17 (10.1) | 37 (10.9) |
| Tacrolimus | 1 (0.6) | 2 (1.2) | 3 (0.9) |

Abbreviations: csDMARD, conventional synthetic disease-modifying antirheumatic drug; PBO, placebo; QD, once daily; UPA, upadacitinib

**SUPPLEMENTARY TABLE 3** Change from baseline at Week 12 in components of ACR

|  | Change from baseline | | Treatment difference | *P* value |
| --- | --- | --- | --- | --- |
|  | **PBO** | **UPA 15 mg QD** |  |  |
| HAQ-DI | –0.18 | –0.62 | –0.44 | < .001 |
| TJC68 | –7.13 | –14.51 | –7.38 | < .001 |
| SJC66 | –4.67 | –8.74 | –4.07 | < .001 |
| PtGA (mm) | –15.24 | –33.41 | –18.17 | < .001 |
| PhGA (mm) | –21.73 | –36.95 | –15.22 | < .001 |
| Patient’s assessment of pain | –15.18 | –35.94 | –20.76 | < .001 |
| hsCRP (mg/L) | –2.49 | –16.75 | –14.26 | < .001 |

Abbreviations: ACR, American College of Rheumatology; HAQ-DI, Health Assessment Questionnaire-Disability Index; hsCRP, high-sensitivity C-reactive protein; PBO, placebo; PhGA, Physician’s Global Assessment of Disease Activity; PtGA, Patient’s Global Assessment of Disease Activity; QD, once daily; SJC66, swollen joint count of 66 joints; TJC86, tender joint count of 68 joints; UPA, upadacitinib

**SUPPLEMENTARY TABLE 4** ACR20 response rate at Week 12 by concomitant csDMARD at baseline

|  | PBO | UPA 15 mg QD | Treatment difference |
| --- | --- | --- | --- |
| Methotrexate alone | 25/71 (35.2) | 57/79 (72.2) | 37 |
| Methotrexate and another csDMARD | 13/41 (31.7) | 25/32 (78.1) | 46.4 |
| csDMARD other than methotrexate | 15/57 (26.3) | 39/57 (68.4) | 42.1 |

Abbreviations: ACR20, ≥20% improvement in American College of Rheumatology criteria; csDMARD, conventional synthetic disease-modifying antirheumatic drug; PBO, placebo; QD, once daily; UPA, upadacitinib

**Supplementary Table 5** Change from baseline in laboratory parameters and frequency of Grade 3 and 4 decreases through Week 12

| Parameter | | PBO + csDMARDs (n = 167) | UPA 15 mg QD + csDMARDs (n = 167) |
| --- | --- | --- | --- |
| Hemoglobin, g/dL | Change from baseline at Week 12, mean (SD)^a^ | 0.7 (7.9) | 2.6 (11.0) |
|  | Grade 3 (70–<80 or decreased 21–<30), n (%) | 1 (0.6) | 2 (1.2) |
|  | Grade 4 (<70 or decreased ≥30), n (%) | 1 (0.6) | 1 (0.6) |
| Lymphocytes, × 10^9^/L | Change from baseline at Week 12, mean (SD)^a^ | 0.0 (0.4) | 0.1 (0.5) |
|  | Grade 3 (0.5–<1.0), n (%) | 17 (10.2) | 16 (9.6) |
|  | Grade 4 (<0.5), n (%) | 1 (0.6) | 1 (0.6) |
| Neutrophils, × 10^9^/L | Change from baseline at Week 12, mean (SD)^a^ | −0.2 (1.8) | −1.2 (2.0) |
|  | Grade 3 (0.5–<1.0), n (%) | 1 (0.6) | 0 |
|  | Grade 4 (<0.5), n (%) | 0 | 0 |
| ALT, U/L | Change from baseline at Week 12, mean (SD)^b^ | 1.7 (15.8) | 2.7 (32.0) |
|  | Grade 3 (3.0–<8.0 × ULN), n (%) | 4 (2.4) | 2 (1.2) |
|  | Grade 4 (>8.0 × ULN), n (%) | 1 (0.6)^d^ | 0 |
| AST, U/L | Change from baseline at Week 12, mean (SD)^c^ | 0.8 (11.7) | 4.6 (17.7) |
|  | Grade 3 (3.0–<8.0 × ULN), n (%) | 2 (1.2) | 2 (1.2) |
|  | Grade 4 (>8.0 × ULN), n (%) | 1 (0.6) | 0 |
|  | Change from baseline at Week 12, mean (SD)^b^ | 3.4 (37.1) | 58.5 (76.2) |
| Creatine kinase, U/L | Grade 3 (>5.0–<10.0 × ULN), n (%) | 0 | 2 (1.2) |
|  | Grade 4 (>10.0 × ULN), n (%) | 0 | 0 |

Abbreviations: ALT, alanine aminotransferase; AST, aspartate aminotransferase; csDMARD, conventional synthetic disease-modifying antirheumatic drug; PBO, placebo; QD, once daily; SD, standard deviation; ULN, upper limit of normal; UPA, upadacitinib

^a^PBO: n = 155, UPA: n = 161.

^b^PBO: n = 154, UPA: n = 159.

^c^PBO: n = 153, UPA: n = 159.

^d^One patient experienced an event of drug-induced liver injury and met Hy’s law criteria.
